# Clinical and societal burden of incident major depressive disorder: A population‐wide cohort study in Stockholm

**DOI:** 10.1111/acps.13414

**Published:** 2022-03-02

**Authors:** Johan Lundberg, Thomas Cars, Sven‐Åke Lööv, Jonas Söderling, Jari Tiihonen, Amy Leval, Anna Gannedahl, Carl Björkholm, Mikael Själin, Clara Hellner

**Affiliations:** ^1^ Centre for Psychiatry Research Department of Clinical Neuroscience Karolinska Institutet Stockholm Health Care Services Region Stockholm Stockholm Sweden; ^2^ Sence Research AB Uppsala Sweden; ^3^ Department of Medical Sciences Uppsala University Uppsala Sweden; ^4^ Stockholm Health Care Services Region Stockholm Stockholm Sweden; ^5^ Epidemiology Division Department of Medicine Solna Karolinska Institutet Stockholm Sweden; ^6^ Department of Forensic Psychiatry University of Eastern Finland Niuvanniemi Hospital Kuopio Finland; ^7^ Neuroscience Center University of Helsinki Helsinki Finland; ^8^ Janssen‐Cilag AB Solna Sweden; ^9^ Department of Medical Epidemiology and Biostatistics Karolinska Institutet Stockholm Sweden

**Keywords:** antidepressant therapy, epidemiology, health resource utilization, major depressive disorder, mortality, observational cohort study, work loss

## Abstract

**Objective:**

Major depressive disorder (MDD) is a highly prevalent condition and a significant contributor to global disability. The vast majority of MDD is handled by primary care, but most real‐life studies on MDD only include data from secondary care. The aim of this study was therefore to estimate the total clinical and societal burden of incident MDD including data from all healthcare levels in a large well‐defined western European healthcare region.

**Methods:**

Population‐wide observational study included healthcare data from Region Stockholm, Sweden's largest region with approximately 2.4 million inhabitants. All patients in Region Stockholm having their first unipolar MDD episode between January 1, 2012, and December 31, 2018, were included. The sample also included matched study population controls. Outcomes were psychiatric and non‐psychiatric comorbid conditions, antidepressant therapy use, healthcare resource utilization, work loss, and all‐cause mortality.

**Results:**

In the study period, 137,822 patients in Region Stockholm were diagnosed with their first unipolar MDD episode. Compared with matched controls, MDD patients had a higher burden of non‐psychiatric and psychiatric comorbid conditions, 3.2 times higher outpatient healthcare resource utilization and 8.6 times more work loss. MDD was also associated with a doubled all‐cause mortality compared with matched controls (HR: 2.2 [95% CI: 2.0–2.4]).

**Conclusions:**

The high mortality, morbidity, healthcare resource utilization, and work loss found in this study confirms that MDD is associated with individual suffering and low functioning leading to substantial costs for patients and society. These findings should motivate additional efforts in improving outcomes for MDD patients.

**Significant Outcomes**

- Patients with incident MDD had 8.6 times more work loss than matched controls.
- All‐cause mortality was 2.2 times higher among patients with MDD compared with matched controls.

**Limitations**

- Coverage of recordings of psychotherapy sessions has increased over the study period, and the true proportion of patients treated with psychotherapy may therefore be underestimated.
- Certain private caregivers (less than 6% of all healthcare visits) may have limited data on some diagnoses.

## 1 INTRODUCTION

Major depressive disorder (MDD) is a highly prevalent condition and a main contributor to global disability,^1^ exceeding even that of cardiovascular diseases.^2^ Recent data from the Global Burden of Disease study show a 50% increase in incident cases of depression from 1990 to 2017,^1^ with a lifetime prevalence between 10% and 20%.^3^ The burden of MDD extends beyond living with depressive symptoms to also influence mortality, for example, MDD is the leading cause of death by suicide worldwide,^2^ with a 20‐fold risk increase compared with healthy individuals.^4^

The presentation of MDD is heterogenous, with notable negative effects on functioning and quality of life.^5^ The disorder is characterized by low mood or sadness, attention deficits, lassitude, pessimism, and in many cases suicidal thoughts or behavior. For many patients, it is a chronic or recurrent illness,^6^, ^7^ and on average, patients with major depression have been estimated to be symptomatic 60% of their lifetime, even when receiving community‐standard antidepressant treatment.^7^

The vast majority of MDD patients are cared for by primary care,^8^, ^9^ but because of the complex nature of the disease, many different specialist care providers are also involved. This makes real‐life studies of depression care that encompass all healthcare levels challenging, with a scarcity of holistic depression studies as a result. Population‐wide studies are necessary to obtain correct estimates of incidence, treatments, comorbidities, and healthcare resource utilization. Real‐life data on treatment outcomes and their relation to current guidelines should inform decision makers on necessary adjustments in, for example, resource allocation within the healthcare system.

Sweden has unique opportunities for observational research because of the country's civic registration system^10^ and the fact that all residents have universal access to healthcare^11^ with a negligible co‐payment for healthcare visits, hospitalizations, and drugs. The public healthcare provider Region Stockholm delivers primary and secondary healthcare to all citizens in the region and data from all healthcare levels are collected in structured databases.^12^ In addition, data can be linked to the Swedish Social Insurance Agency (SSIA) registries, providing data on work loss, which includes a medical and insurance‐based assessment.

We have established a large population‐based cohort (referred to as the Stockholm MDD Cohort) comprising all patients who were diagnosed with MDD in Region Stockholm between 2010 and 2018. The aim of the current study was to estimate the clinical and societal burden of incident MDD to ultimately guide improvements in patient care.

## 2 METHODS

This is a population‐wide observational study comprising all patients with incident MDD in Region Stockholm which, with its 2.4 million inhabitants, accounts for approximately 24% of the Swedish population.^13^ The study was approved by the regional ethics committee, Stockholm, Sweden (No: 2018/546‐31) and registered at ENCePP (www.encepp.eu, EU PAS Register Number: 25646).

### 2.1 Data sources

To capture a complete overview of drug utilization, comorbid conditions, healthcare resource utilization, and work loss for patients diagnosed with MDD, data were linked using the following three data sources: (1) the Stockholm regional healthcare data warehouse (VAL),^12^ (2) Electronic Medical Records (EMRs) in the Region of Stockholm,^14^ and (3) the registries held at the Swedish Social Insurance Agency (SSIA).^15^ All data were linked using the personal identity number unique to each Swedish citizen^10^ and analyzed in a pseudonymized format.

### 2.2 Study participants

All patients in Region Stockholm having their first (ever) MDD episode, incident major depressive disorder (ICD10: F32, F33), between January 1, 2012 and December 31, 2018 were included. Index date was date of first recorded MDD diagnosis. In order to only include patients with unipolar MDD, we excluded patients having a history of psychosis (ICD10: F20–F29), bipolar disorder (ICD10: F31), manic episode (ICD10: F30), or dementia (ICD10: F00–F03). Furthermore, in order to obtain information on comorbid conditions, healthcare utilization, and drug utilization before first MDD episode, we excluded patients residing in Region Stockholm ≤12 months. The final study population also included population controls (individuals with no recordings of depression, intentional self‐harm or antidepressant therapy) matched (with replacement) by age (within 2 years), sex, and municipality. Matched controls were given the same index date as the matched case. The exclusion criteria applied to patients with MDD were also applied to matched controls.

### 2.3 Definitions

#### 2.3.1 MDD episode

We established a definition as a proxy for the duration of the MDD episode (or rather, the duration of healthcare contacts related to MDD). This was operationally defined based on recorded activities related to MDD:

- For each patient, we selected the patient's first (ever) recorded MDD diagnosis (ICD10: F32‐Depressive episode and F33‐Recurrent depressive episode), initiating the first recorded MDD episode (i.e., the index date).
- For each patient, we analyzed the time from the first recorded MDD diagnosis to subsequently recorded events related to depression. If the time interval between the recording of depressive events was ≤365 days, the episode was categorized as ongoing while if the time interval was >365 days, the episode was categorized as closed at the date of the last recorded depressive event. As depressive events, we allowed (1) recording of MDD diagnoses (recorded in any healthcare level). (2) filled prescriptions of antidepressants (AD; ATC: N06A), and add‐on medication for depression (lithium, risperidone, olanzapine, aripiprazole, and quetiapine [>100 mg]), electroconvulsive therapy (ECT), repetitive transcranial magnetic stimulation (rTMS), or treatment with psychotherapy. If the last recorded depressive event was a dispensation of either AD or add‐on medication, we extended the episode with the number of dispensed tablets (a maximum of 100 days was added).
- When an episode is categorized as closed, a subsequent recording of an MDD diagnosis code would initiate the start of a new MDD episode.

In a sensitivity analysis, we decreased the time interval between the recording of depressive events to 180 days (6 months).

### 2.4 Covariates

For all study participants, we included information on age, sex, healthcare level where the patients were initially diagnosed, outpatient and inpatient healthcare utilization, work loss (sick leave and disability pension), and history of psychiatric and non‐psychiatric comorbid conditions. For each study participant, we also included treatment with the following antidepressant therapies: AD, add‐on medication, ECT, rTMS, and psychotherapy. From EMRs, we also extracted information on body mass index (BMI) and smoking habits. All codes for defining variables are presented in Table S1.

### 2.5 Statistical analyses

Numbers and proportions were calculated for categorical variables and means, medians, standard deviations (SD), and interquartile ranges (IQR) were reported for continuous variables.

For all patients with incident MDD, we analyzed antidepressant therapy (AD, add‐on medication, ECT, rTMS, and psychotherapy), healthcare utilization, work loss, and psychiatric comorbid conditions from 12 months before and up to 12 months after index date. For study population controls, we analyzed healthcare utilization and work loss. In these analyses, patients were censored at the first instance of emigration from Region Stockholm, death, or any of the exclusion criteria (psychosis, dementia, manic episode, or bipolar disorder). Duration of incident MDD episode was calculated as the time from the start of the episode until the end of the episode and presented in a Kaplan–Meier plot. Analysis on episode duration was only performed for patients with at least 1 year of follow‐up to enable assignment of an episode end date.

#### 2.5.1 Antidepressant therapy

For each patient, and for each month, we analyzed all filled prescriptions (AD and add‐on medication) and recorded clinical procedure codes (ECT, rTMS, and psychotherapy) of antidepressant therapy and calculated the proportion of patients with ongoing antidepressant therapy each month (−12 to +12 months from index). Ongoing treatment of ECT, rTMS, or psychotherapy required patients to have at least one recording of a clinical procedure code for that procedure that month. Equally, actively treated with AD or add‐on medication required each patient to have at least one pharmacy dispensation of AD or add‐on medication that month or covered with medical supply from a previous dispensation (calculated as the number of days supplied plus an additional 25% of medication supply to allow variation in adherence).^16^ For antidepressant therapy, we also calculated the cumulative proportion of patients treated each month, starting from 12 months before index. In this analysis, patients were included the first month they were treated (i.e., starting from 12 months before index) and carried forward in the analysis even if they terminated the treatment.

#### 2.5.2 Psychiatric comorbid conditions

The cumulative proportion of psychiatric comorbid conditions was calculated per month (−12 to +12 months from index). Patients were included the first month they were diagnosed with the psychiatric comorbid condition and carried forward in the analysis. This analysis allowed inclusion of diagnoses recorded within the past 5 years before index.

#### 2.5.3 Healthcare utilization and work loss

Healthcare utilization was presented as the mean number of outpatient visits and inpatient bed days per month (−12 to +12 months from index). For outpatient visits, all in‐person physician visits (visits where the patient met a physician) were included and presented for the following three healthcare levels: (1) all outpatient physician visits, (2) physician visits in primary care, and (3) physician visits in psychiatric care. Inpatient healthcare utilization was presented as the mean number of bed days in (1) all inpatient care, (2) psychiatric inpatient care, and (3) non‐psychiatric inpatient care.

Work loss was described as the mean number of work loss days per month (−12 to +12 months from index) and presented as (1) total work loss, (2) sick leave, and (3) disability pension. Analyses on work loss were performed on individuals with an age ≥20 years and ≤64 years, and sick leave episodes with a duration shorter than 14 days were not included since these are paid by the employer.

In the annual analyses (Table 2) on healthcare utilization and work loss, each individual was assigned a weight based on the time of follow‐up after index (weight_12 month follow‐up after index _= 1). Weighted means and 95% confidence intervals were calculated, and in order to account for the clustering structure of data, we used robust standard errors.

#### 2.5.4 Referrals between healthcare levels

Referral patterns over time were analyzed and presented in an alluvial (“flow”) diagram including primary care, psychiatric care (outpatient and inpatient), and other healthcare levels. Analyses were done at 3‐month intervals to demonstrate the flow between healthcare levels from index date (first MDD diagnosis) to 12 months after index. Categorization was based on the healthcare level for the most recently recorded event related to MDD (see section 2.3.1) before each 3‐month interval timepoint. For example, if a patient initially was diagnosed in primary care and later had a recorded depressive event in psychiatric care just before 3 months after index, that patient is presented in the alluvial diagram as a “flow” from primary care (index) to psychiatric care (3 months).

#### 2.5.5 All‐cause mortality

Associations between patients with incident MDD and matched controls on all‐cause mortality were assessed using Cox proportional hazards models. Individuals were followed from index until the first instance of death, emigration from Stockholm, any of the exclusion criteria (psychosis, dementia, manic episode, or bipolar disorder) or end of follow‐up (December 31^st^, 2018).

All data management and analyses were carried out using R (version 3.6.0).^17^

## 3 RESULTS

Between January 1, 2012, and December 31, 2018, 137 822 patients in Region Stockholm had an incident unipolar MDD episode (Figure S1). A study population control was possible to match to 135 575 (98.4%) patients with incident MDD.

The mean (SD) age at incident MDD was 41.3 (19.4) years, and 63.2% were women (Table 1). The majority (68.2%) of incident MDD episodes were diagnosed in primary care and 27.3% in psychiatric care. The median duration of incident MDD treatment episodes was estimated to 398 days (95% CI: 392–403; Figure S2). In the sensitivity analysis, reducing the interval allowed between depressive events within an episode to 6 months decreased the median duration to 270 days (95% CI: 266–274).

**TABLE 1:** Baseline characteristics for all first MDD episodes in Region Stockholm between 2012 and 2018 and matched controls

	Incident MDD episodes	Matched controls
*N*	137,822	135,575
Demographics		
Age (at index)		
Mean (SD)	41.3 (19.4)	41.3 (19.3)
Median (IQR)	38.0 (26.0–54.0)	38.0 (26.0–54.0)
<18	11,065 (8.0%)	11,000 (8.1%)
18–24	19,184 (13.9%)	18,452 (13.6%)
≥25	107,573 (78.1%)	106,123 (78.3%)
Sex (% women)	87,071 (63.2%)	85,733 (63.2%)
Healthcare level at index (time of the first recorded MDD diagnosis)		
Primary care	93,927 (68.2%)	n/a
Psychiatric care	37,611 (27.3%)	n/a
Other healthcare levels	6284 (4.6%)	n/a
Clinical measurements		
Body Mass Index		
Mean (SD)	25.3 (5.7)	25.4 (5.3)
Median (IQR)	24.4 (21.3–28.3)	24.5 (21.7–28.2)
Missing (%)	90,009 (65.3%)	103,839 (76.6%)
Smoking habits^a^		
Smoker (*N*, %)	7690 (18.7%)	3148 (10.8%)
Non‐smoker (*N*, %)	26,741 (65.1%)	21,498 (73.8%)
Former smoker (*N*, %)	6643 (16.2%)	4488 (15.4%)
Missing (%)	96,748 (70.2%)	106,441 (78.5%)
Psychiatric comorbid conditions^b^		
Anxiety	37,090 (26.9%)	3382 (2.5%)
Stress (excl. posttraumatic stress disorder)	29,563 (21.5%)	4434 (3.3%)
Sleep disorders	20,178 (14.6%)	4352 (3.2%)
Disorders because of substance use	9818 (7.1%)	1734 (1.3%)
Disorders because of alcohol use *(also included in substance use)*	6342 (4.6%)	1210 (0.9%)
Obsessive compulsive disorder	2188 (1.6%)	152 (0.1%)
Hyperkinetic disorders	5310 (3.9%)	1414 (1.0%)
Autism spectrum disorders	1865 (1.4%)	412 (0.3%)
Personality disorders	1168 (0.8%)	41 (0.0%)
Intentional self‐harm	1889 (1.4%)	n/a
Non‐psychiatric comorbid conditions^b^		
Cardiovascular comorbidity	10,772 (7.8%)	6674 (4.9%)
Hypertension	21,098 (15.3%)	16,295 (12.0%)
Diabetes Mellitus type II	5611 (4.1%)	3970 (2.9%)
Hypothyroidism	7604 (5.5%)	5347 (3.9%)
Inflammatory bowel disease	1319 (1.0%)	889 (0.7%)
Rheumatoid arthritis	952 (0.7%)	655 (0.5%)
Antidepressant treatment (treatment within 12 months before the first MDD diagnosis)		
Antidepressants (ATC: N06A – Antidepressants)	36,111 (26.2%)	n/a
Add‐on medication (lithium, risperidone, olanzapine, aripiprazole, and quetiapine [>100 mg])	1355 (1.0%)	n/a
ECT	14 (0.0%)	n/a
Psychotherapy	9521 (6.9%)	924 (0.7%)

Note

All codes for defining comorbid conditions are presented in Table S1.

^a^ Percentages are calculated based on non‐missing values

^b^ We included comorbid conditions recorded 5 years prior to index

The all‐cause mortality rate was 8.6 deaths per 1000 person years at risk, based on 5747 deaths over a median (IQR) of 2.2 (1.2–3.1) years of follow‐up. MDD was associated with a higher all‐cause mortality (HR: 2.2 [95% CI: 2.0–2.4]) compared with matched controls.

**FIGURE 1:**
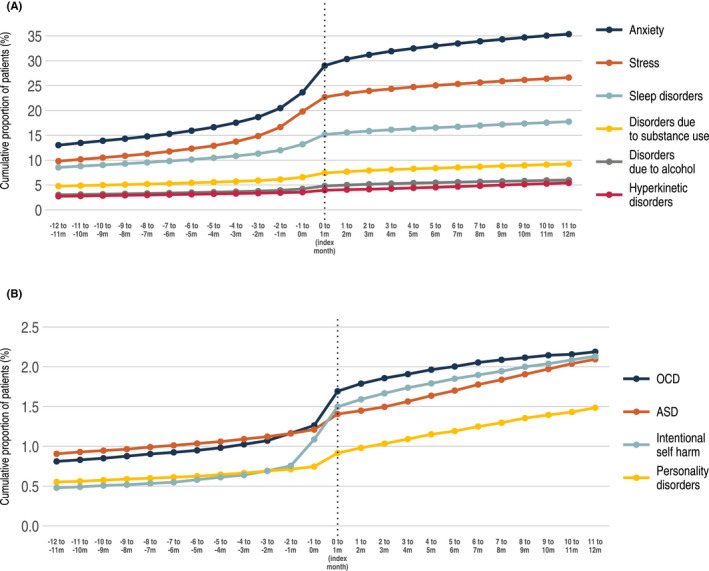
Cumulative proportion of psychiatric comorbid conditions among patients with incident MDD 12 months before and 12 months after the first MDD diagnosis. (A, B) The index month corresponds to the month when the patient received their first MDD diagnosis. Disorder due to alcohol is also included in the category substance use (A)

Of all patients with incident MDD, 26.9% had documented anxiety, 21.5% stress and 14.6% sleep disorders (Table 1) at the time of their first MDD diagnosis. For anxiety and stress, we observed a gradual increase in the prevalence of these conditions over time up to the time of the first MDD episode, with a doubling from 12 months before index to the index month (Figure 1A). Compared with the matched controls, we also observed a higher prevalence of all analyzed non‐psychiatric comorbid conditions among patients with incident MDD (Table 1).

**FIGURE 2:**
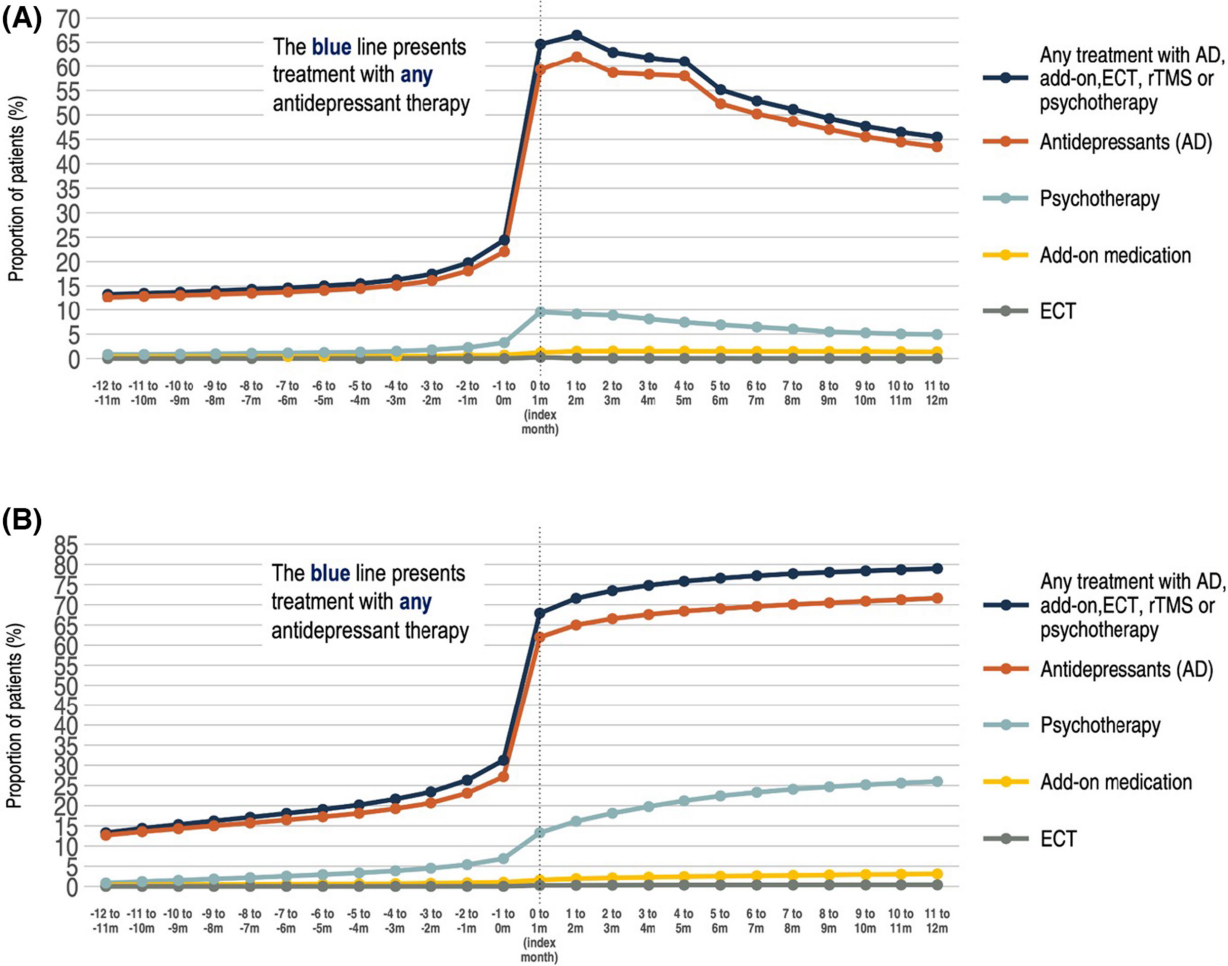
Proportion of antidepressant treatment per month among all patients with incident MDD 12 months before and 12 months after index MDD diagnosis. (A) The proportion of patients with ongoing antidepressant therapy per month starting from 12 months before index. (B) The cumulative proportion of patients treated with antidepressant therapy starting from 12 months before index. In the cumulative analysis, patients are included the first month they are treated and carried forward in the analysis even if they terminated the treatment

A gradual increase in the proportion of patients receiving antidepressant therapy was observed. At 12 months before index, 13.3% of the patients were treated with either AD, add‐on medication, ECT, rTMS, or psychotherapy, increasing to 24.4% the month before index (Figure 2A). In the index month (the calendar month when the first MDD diagnosis was recorded), 64.6% were actively treated with antidepressant therapy. Within the time period of 12 months before and 12 months after index, 79.0% of patients with incident MDD had been treated with at least one antidepressant therapy (Figure 2B).

**FIGURE 3:**
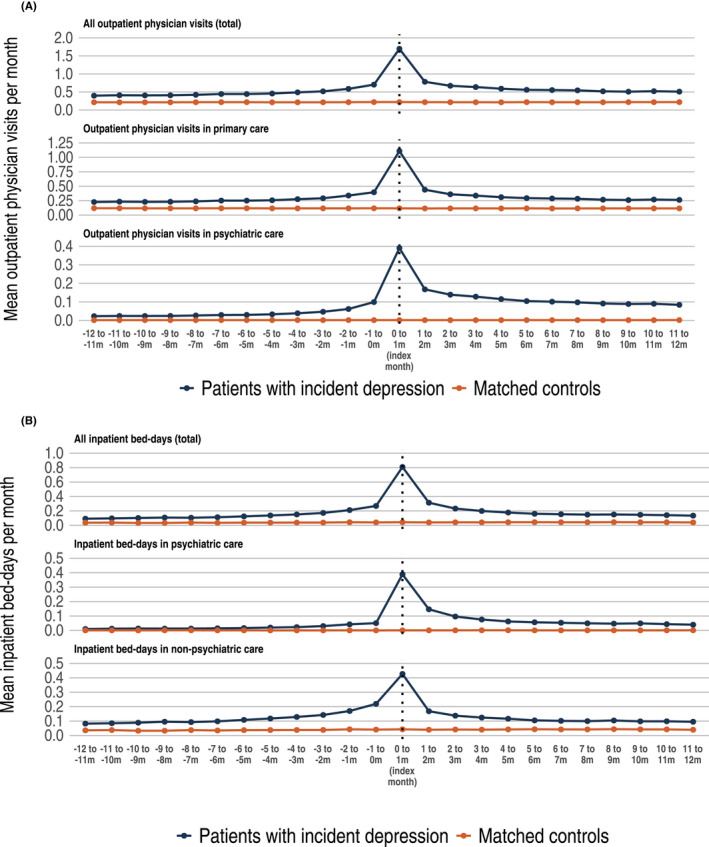
Healthcare resource utilization among patients with incident MDD compared with matched study population controls. (A) The mean number of outpatient physician visits per months from 12 months before index up to 12 months after index. Upper panel: mean number of outpatient physician visits in any healthcare level (total). Middle panel: mean number of outpatient physician visits in primary care. Lower panel: mean number of outpatient physician visits in psychiatric care. (B) The mean number of inpatient bed days per months from 12 months before index up to 12 months after index. Upper panel: mean number of inpatient bed days in any health care (total). Middle panel: mean number of inpatient bed days in psychiatric care. Lower panel: Mean number of inpatient bed days in non‐psychiatric care. Please note that the y‐axis has different scales

**FIGURE 4:**
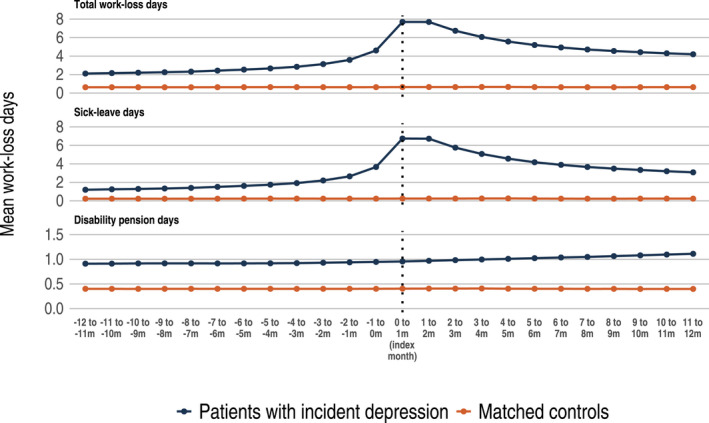
Lost workdays among patients with incident MDD compared with matched study population controls. The mean number of work loss days from 12 months before index up to 12 months after index. Upper panel: mean number of total work loss days. Middle panel: mean number of sick leave days. Lower panel: mean number of days with disability pension

Healthcare resource utilization (HRU) is presented in Figure 3A,B and in Table 2. For both outpatient and inpatient visits, a substantial peak was observed during the index month. Mean outpatient physician visits 12 months before and 12 months after index were 2.2 and 3.2 times higher among patients with incident MDD, compared with controls. A similar pattern was observed for work loss, where total work loss days among patients with incident MDD compared with population controls were 4.3 and 8.6 times higher 12 months before and 12 months after index (Figure 4). Work loss was mainly explained by the increase in number of sick leave days, while days of disability pension remained stable over the follow‐up period.

**TABLE 2:** Healthcare resource utilization and work loss for patients with incident MDD compared with matched study population controls

	Mean healthcare resource utilization and work loss 12 months before index		Mean healthcare resource utilization and work loss 12 months after index (incl. index date)	
	Incident MDD episodes	Matched study population controls	Incident MDD episodes	Matched study population controls
Outpatient physician visits (Mean, 95% CI)				
Total	5.68 (5.65–5.71)	2.58 (2.55–2.62)	7.90 (7.86–7.93)	2.48 (2.45–2.51)
Primary care	3.21 (3.19–3.24)	1.41 (1.39–1.43)	4.40 (4.37–4.42)	1.33 (1.31–1.35)
Psychiatric care	0.46 (0.45–0.47)	0.021 (0.019–0.023)	1.57 (1.55–1.58)	0.020 (0.018–0.022)
Inpatient bed days (Mean, 95% CI)				
Total	1.68 (1.64–1.72)	0.45 (0.42–0.48)	2.60 (2.53–2.66)	0.48 (0.45–0.50)
Psychiatric care	0.26 (0.24–0.28)	0.004 (0.002–0.007)	1.07 (1.03–1.12)	0.007 (0.003–0.012)
Non‐psychiatric care	1.43 (1.39–1.47)	0.45 (0.42–0.48)	1.53 (1.49–1.58)	0.47 (0.44–0.49)
Work loss days (Mean) (Mean, 95% CI)				
Total days of work loss	32.88 (32.37–33.38)	7.65 (7.26–8.05)	64.80 (64.10–65.50)	7.47 (7.07–7.88)
Sick‐leave days	21.81 (21.43–22.18)	2.83 (2.66–3.00)	52.67 (52.05–53.29)	2.80 (2.63–2.98)
Days with disability pension	11.07 (10.71–11.43)	4.82 (4.46–5.18)	12.12 (11.73–12.51)	4.67 (4.31–5.04)

Note

Table 2 presents healthcare resource utilization and work loss 12 months before and 12 months after index. Patients with incident MDD are compared with matched controls and given the same index date as the case.

**FIGURE 5:**
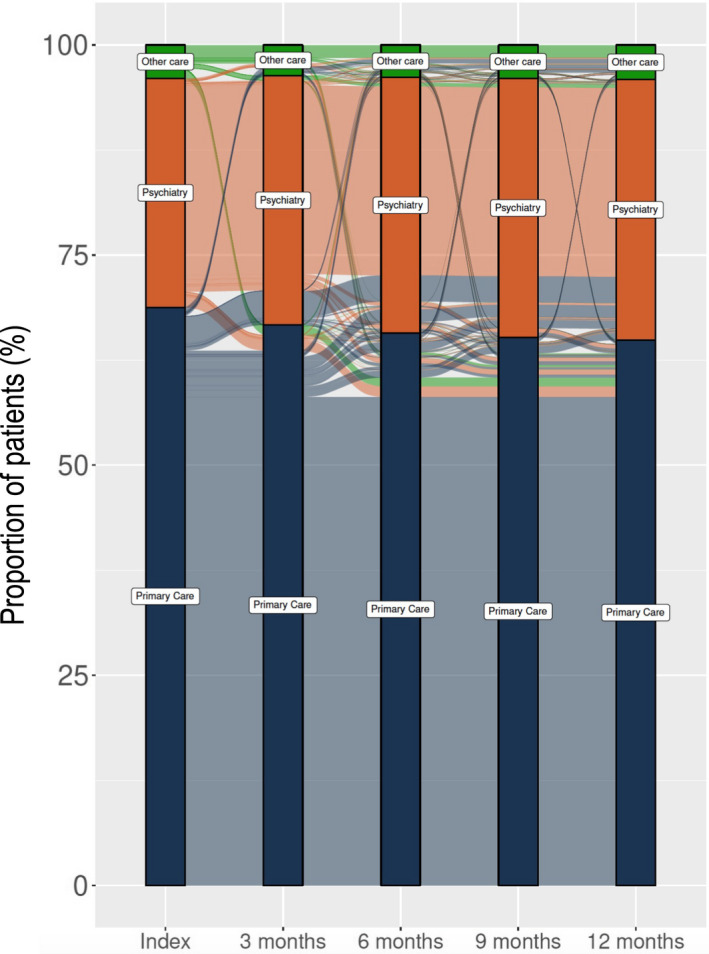
Mobility between healthcare levels for patients with incident MDD within 12 months after index. The index bar presents the healthcare level where the MDD episode was initiated, and this flow diagram further presents in which healthcare level patients are managed 3,6,9, and 12 months after index

Figure 5 presents referrals between healthcare level for patients with incident MDD within 12 months after index. The vast majority of patients initially diagnosed with MDD within primary care were still treated in primary care 12 months after the first diagnosis. Similarly, those with an initial diagnosis in specialist care remained, and overall we observed a limited mobility between healthcare levels during 12 months after index.

## 4 DISCUSSION

In this population‐wide observational study, we show that patients with incident MDD have high somatic and psychiatric comorbidity, a doubled risk of death, tripled number of healthcare visits, and approximately nine times more workdays lost to disease compared with matched controls.

As expected, the majority of patients were treated in primary care. However, we observed low mobility between healthcare levels, that is, from primary to psychiatric care and back. In this study, 13.8% of those initially seen in primary care were handled in psychiatric care related to MDD within the first year after diagnosis, which can be compared with previously reported referral rates ranging from 16 to 58%.^18^, ^19^, ^20^ In addition to methodological differences in the studies hampering direct comparisons, country‐specific differences in how healthcare systems are organized and how care is coordinated between primary and secondary care will also affect observed referral rates. Further studies are needed to investigate if and how patient referrals could be optimized.

The literature of the duration of MDD episodes is limited and seldom includes both inpatient and outpatient care for an entire geographic region. One observational study from the United States reported an MDD duration of approximately 300 days^21^ using data from claims databases. We found that decreasing the allowed time interval between the episodes from 365 to 180 days substantially reduced the episode duration (from 398 days to 270 days). Since we were not able to confirm the patients’ symptom severity using, for example, clinical ratings scales, our definition is based on recorded activities and treatment patterns related to depression rather than the actual symptomatology *per se*. It will rather be an estimate of the duration of the total healthcare contacts related to the MDD episode and might overestimate the actual length of the active MDD episode.

Depression is often accompanied by other psychiatric disorders, and the association between depression and anxiety is well established.^22^ Studies have reported that approximately 50%–60% of individuals with depression also describe a history of anxiety disorders.^23^ For all analyzed psychiatric comorbid conditions, we observed a gradual increase in the cumulative prevalence over time before the first diagnosis of MDD, where the most striking increases were observed for anxiety and stress. The data do not allow us to identify any cause for this pattern, but one possible hypothesis is the substantial overlap in symptoms between many of the disorders that increase in prevalence; something that is particularly true for anxiety, stress, and depressive disorders.

Unsurprisingly, most patients were at some point prescribed AD treatment following their MDD diagnosis (80% at 12 months’ post‐index). Around one in four patients were prescribed AD treatment within the year prior to their first MDD diagnosis, which is likely explained by the treatment of preexisting comorbid disorders, which are often also treated with ADs (i.e., anxiety disorders) and by the gradual onset of MDD. According to national and regional guidelines, psychotherapy is recommended as the first‐line treatment for mild‐to‐moderate depression. Approximately 7% of all patients with incident MDD had a record of beeing treated with psychotherapy for any indication within 1 year before their first MDD diagnosis. In addition, around one quarter of patients had a recorded psychotherapy treatment session at some point during the follow‐up period.

Current treatment guidelines typically recommend a sequenced approach to depression treatment when remission or significant improvement is not met within four weeks of treatment initiation.^24^, ^25^ Our data show that treatments, such as augmentation medication, are rarely prescribed within 12 months of the first MDD diagnosis. This could partly be explained by the fact that the majority of patients included in our cohort are managed in primary care and are therefore likely suffering from less severe forms of depression not in need of augmentation treatment. In addition, 12 months may not be considered a long enough episode duration for augmentation treatments to be prescribed, for the first‐episode patients.

All‐cause mortality was twice as high in the MDD group than in population controls. Although we did not have access to causes of death, rates of intentional self‐harm increases after, and around, the time of the MDD‐diagnosis, indicating that suicide could be a contributing factor to the excess mortality. A meta‐analysis^26^ of 293 studies with a total of close to 2 million subjects showed that the overall MDD‐associated excess mortality was increased by 52% and did not appear to differ in patient groups with or without comorbidities (such as cancer and heart disease; with the exception of chronic obstructive pulmonary disease), indicating that depression is an independent risk factor. The slightly lower mortality risk reported in the meta‐analysis compared with that of the present study might be explained by the fact that we have selected relatively healthy controls, that is, individuals free of depression, intentional self‐harm, or antidepressant therapy. This further underscores the impact of these conditions on the general health of the population.

Work loss is an important marker for depression disability, since information is available for the whole population of working age. Although sick leave is influenced by local rules and regulations, it is a measure of doctor‐perceived overall function of the patient, that has to be approved by the SSIA. Previous studies have repeatedly shown that depression is associated with very high costs to healthcare and the society, where indirect costs accounts for up to 90%.^27^, ^28^, ^29^ We observed a substantial increase in the number of workdays lost in the MDD group, which was mainly driven by sick leave (as opposed to disability pension). We observed a sharp increase in the mean number of workdays lost around time of the first MDD diagnosis, followed by a slow decline for the following 12 months. Interestingly, while the HRU appeared to decline to pre‐diagnosis levels a few months after index date, sick leave did not return to pre‐diagnosis levels within 12 months after the first MDD diagnosis. Twelve months following the first MDD diagnosis, the aggregated work loss was almost nine times higher in the MDD group compared with matched population controls. The mean total work loss was 65 days in the first year after an MDD diagnosis. This is substantially higher than in previous reports.^30^, ^31^, ^32^ Differences between countries in terms of labor and sick leave laws may explain much of these differences; Swedish absenteeism may to some degree correspond to presenteeism in other countries. Compared with other reports, the differences may also be explained by the fact that we have established a cohort of patients newly diagnosed with MDD where we observe a higher rate of work loss closer to the first MDD diagnosis, followed by a gradual decline. In our analysis, we also observe a large interindividual variation in work loss. Thus, future studies should aim to gain knowledge of factors that contribute to the long‐term work loss among patients with depression to enable interventions specifically for this group.

The organization of healthcare systems influences the setting in which depression patients are managed and analyzing data from all healthcare levels increases the generalizability of the findings to other healthcare systems. In the present study, we were able to describe the significant impact of incident MDD through measures of episode duration, comorbidities, intentional self‐harm, HRU, work loss, and mortality in a total population of 2.4 million people including data from all healthcare levels collected over 7 years—the most extensive and complete survey of a large population to date. The significant impact on all measures coupled with the high incidence rates of MDD confirms that MDD is associated with a high burden to patients and to society. These findings should motivate substantial efforts in improving treatment outcomes in this patient group.

## 5 STRENGTHS AND LIMITATIONS

We have extracted and linked data from both administrative data sources and EMRs to build a comprehensive overview of healthcare utilization, comorbid conditions, treatments, and work loss of depression in a large well‐defined healthcare region. Compared with other observational studies on MDD, the main strength in this study lies in that we have included data from all healthcare levels including primary and secondary care.

This data source includes data on all healthcare in Stockholm financed by Region Stockholm with complete data on inpatient health care. For outpatient care, data on diagnosis are lacking from certain private caregivers, which account for around 6% of all outpatient physician visits, which may lead to an underestimation of outpatient healthcare utilization among patient with incident MDD. According to changes in contracts with caregivers in the Region Stockholm, the coverage of recordings of psychotherapy sessions has increased over time and the true proportion of patients treated with psychotherapy may be underestimated.

## RESEARCH PROJECT ORGANIZATION AND ROLES AND FUNDING

This research project is part of a framework aimed to facilitate research collaborations between the public healthcare authorities in Stockholm County, Sweden, and research‐based companies. All pharmaceutical companies in Sweden were invited to participate in this depression research program via the pharmaceutical industry trade association. Region Stockholm was the initiator of this research project and governed all research data. Region Stockholm further supported this project with scientific and clinical expertise. In this research program, Janssen‐Cilag AB Sweden participated and in addition to providing scientific expertise, Janssen‐Cilag AB also supported the analytic work for this project via an external company in biostatistics (Sence Research AB). No financial transfers were made between Janssen‐Cilag AB and Region Stockholm.

### PEER REVIEW

The peer review history for this article is available at https://publons.com/publon/10.1111/acps.13414.

## Supporting information

Supplementary Material

## Data Availability

Data for this research project will be available upon reasonable request after performed legal assessment to ensure that confidentiality of the data will be maintained.
