# Supplementary material for: Clinical and societal burden of incident major depressive disorder: A population‐wide cohort study in Stockholm
Source: Acta Psychiatr Scand. 2022 Mar 2;146(1):51–63. doi: 10.1111/acps.13414 (PMC9310720; doi:10.1111/acps.13414)
Supplement: Supplementary file 1 — Supplementary Material [file ACPS-146-51-s001.docx]

# Supplementary tables

Supplementary table 1 - Definition of covariates

| **Condition** | **Included codes** |
| --- | --- |
| *Psychiatric conditions* |  |
| MDD diagnosis | ICD10:  F32 – Depressive episode  F33 - Recurrent depressive disorder |
| Psychosis | ICD10:  F20-F29 Schizophrenia, schizotypal and delusional disorders |
| Manic episode | ICD10:  F30 - Manic episode |
| Bipolar disorders | ICD10:  F31 - Bipolar affective disorder |
| Dementia | ICD10:  F00 – Dementia in Alzheimer disease  F01 – Vascular dementia  F02 – Dementia in other diseases classified elsewhere  F03 – Unspecified dementia |
| Anxiety | ICD10:  F41– Other anxiety disorders |
| OCD | ICD10:  F42– Obsessive compulsive disorder |
| Stress | ICD10:  F43– Reaction to severe stress, and adjustment disorders (excl. PTSD F43.1) |
| Disorders due to substance use | ICD10:  F10-F19 - Mental and behavioral disorders due to psychoactive substance use |
| Disorders due to alcohol use | ICD10:  F10 - Mental and behavioral disorders due to use of alcohol |
| Personality disorders | ICD10:  F60 - Specific personality disorders |
| Hyperkinetic disorders | ICD10:  F90 - Hyperkinetic disorders |
| ASD – Autism spectrum disorders | ICD10:  F840 - Childhood autism  F841 - Atypical autism  F845 - Asperger syndrome |
| Intentional self-harm | ATC:  X60-X84 – Intentional self-harm |
| *Non-psychiatric conditions* |  |
| Cardiovascular disease | ICD10:  I20-I25, I50, I110, I42 (excl I42.1 and I42.2), I43, I48, I60-I64, G45, I70-I72, I73.1, I73.9, I74, I77.3, I77.6, I77.8, I79 |
| Hypertension | ICD10:  I10-I15 |
| Diabetes mellitus type II | ICD10:  E11 - Diabetes mellitus type II |
| Rheumatoid arthritis | ICD10:  M05 - Seropositive rheumatoid arthritis  M06 - Other rheumatoid arthritis |
| Inflammatory bowel disease | ICD10:  K50 – Crohn disease [regional enteritis]  K51 - Ulcerative colitis |
| Hypothyroidism | ICD10:  E03 - Other hypothyroidism |
| *Antidepressant therapy* |  |
| AD | ATC:  N06A - Antidepressants |
| Add-on medication | ATC:  N05AN01 - Lithium  N05AX08 - Risperidone  N05AH03 - Olanzapine  N05AX12 - Aripiprazole  N05AH04 - Quetiapine (>100 mg) |
| ECT | Clinical procedure codes:  DA006 – ECT, unspecified  DA024 - ECT, unilateral  DA025 - ECT, bilateral |
| rTMS | Clinical procedure codes:  DU050 - Repetitive Transcranial Magnetic Stimulation |
| Psychotherapy | Clinical procedure codes:  DU008 – Psychodynamic therapy  DU009 – Psychotherapy, other  DU010 – Psychotherapy, cognitive  DU011 – Psychotherapy, CBT  DU013 – Psychotherapy, MBT  DU020 – Psychotherapy, systemic  DU021- Psychotherapy, DBT  DU022 – Psychotherapy, IPT  DU023 - Psychopedagogical |

# Supplementary figures

**Supplementary figure 1 – Flow chart for the derivation of the study population**


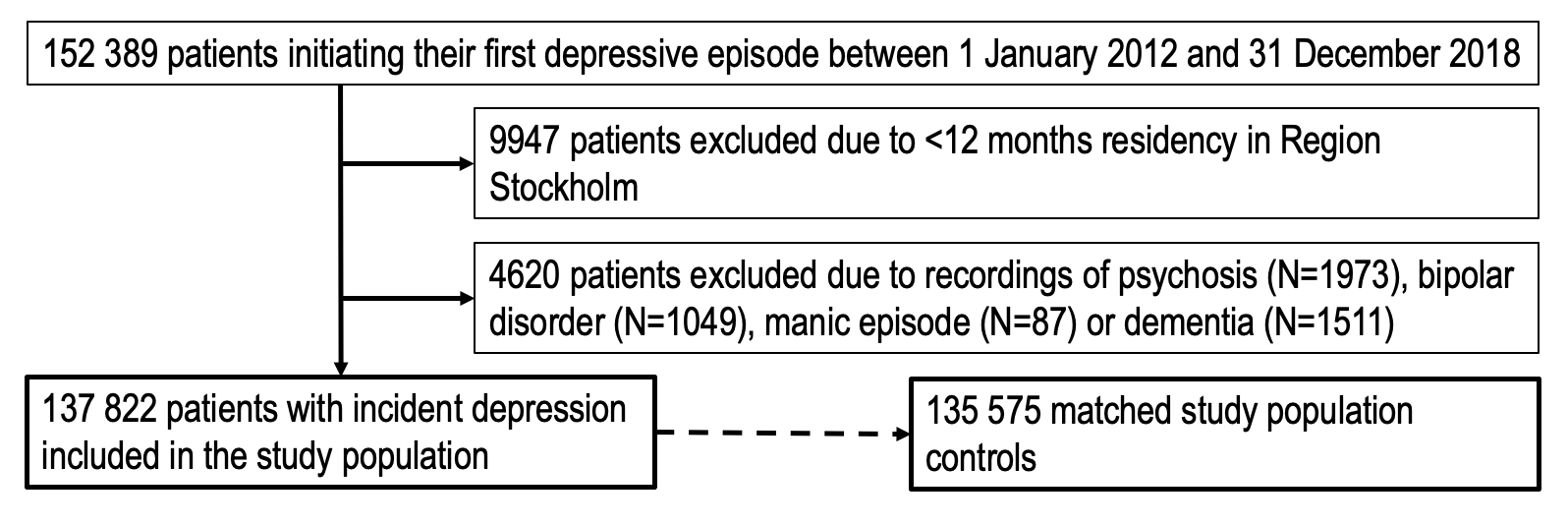


**Supplementary figure 2 – Duration of incident (first) MDD episode**


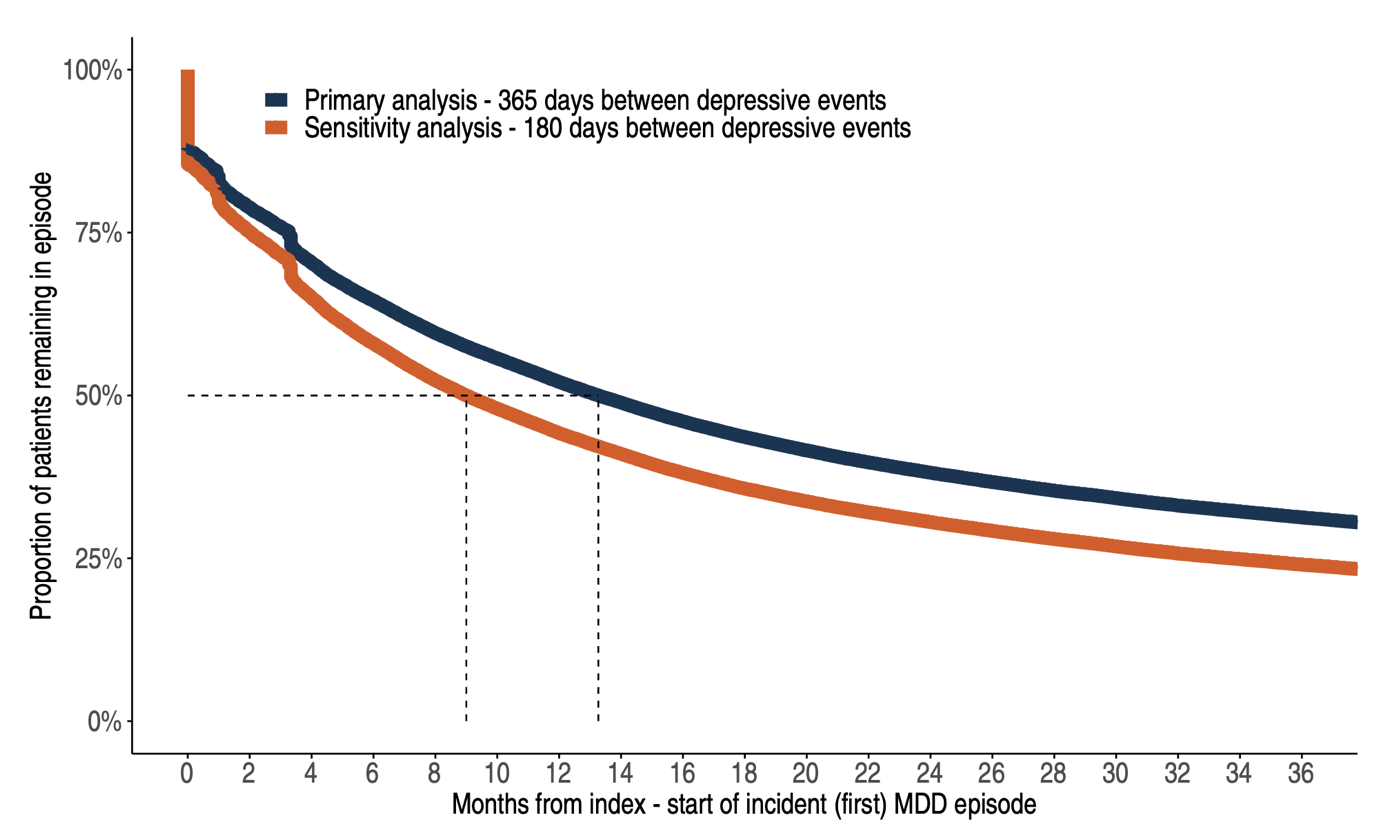


The Kaplan-Meier presents the proportion of patients remaining in their incident (first) MDD episode over time. The dotted line presents the median duration of the episode. If an episode ends with a dispensation of AD or add-on medication, we extend the episode according to the number of dispensed tablets. A maximum of 100 days was added. The most commonly dispensed packages are 30 and 100 tablets; therefore, we observe a “drop” at 30 and 100 days in the Kaplan-Meier curve.
